# Elevation of Metrnβ and Its Association with Disease Activity in Systemic Lupus Erythematosus

**DOI:** 10.3390/ijms241713607

**Published:** 2023-09-02

**Authors:** Chen Zhang, Shijie Cai, Ying Li, Xiaoyan Xu, Yonghui Liu, Huaiyu Qiao, Chun-Kwok Wong, Guoqiu Wu, Hui Jin, Xun Gao

**Affiliations:** 1 Center of Clinical Laboratory Medicine, Zhongda Hospital, Southeast University, Nanjing 210009, China; 2 Department of Epidemiology and Health Statistics, School of Public Health, Southeast University, Nanjing 210009, China; 3 Department of Laboratory Medicine, Medical School of Southeast University, Nanjing 210009, China; 4 Department of Rheumatology, Zhongda Hospital, Southeast University, Nanjing 210009, China; 5 Department of Chemical Pathology, Prince of Wales Hospital, The Chinese University of Hong Kong, Hong Kong, China; 6 Key Laboratory of Environmental Medicine Engineering, Ministry of Education, School of Public Health, Southeast University, Nanjing 210009, China

**Keywords:** Metrnβ, ILC2, pathogenesis, systemic lupus erythematosus

## Abstract

Systemic lupus erythematosus (SLE) is an auto-immune disease, the pathogenesis of which remains to be fully addressed. Metrnβ is a novel cytokine involved in the pathogenesis of inflammatory disease, but its regulatory roles in SLE are unclear. We aimed to comprehensively investigate the clinical value of Metrnβ in SLE. A massive elevation of circulating Metrnβ levels was observed in SLE, and patients with an active phase displayed higher Metrnβ concentrations than those with inactive phases. Additionally, we found that Metrnβ expression was positively correlated with clinical indicators of SLE. Longitudinal cytokine and chemokine profiles revealed a disturbed immune response in SLE, with high activity profiles displayed severe pathogenic inflammation, and a positive correlation of the serum Metrnβ with CXCL9, IL10, IL18 and IL1RA was observed as well. Moreover, Metrnβ expressions exhibited an inverse correlation with Treg and B10. Of note, a significant decrease of ILC2 was found in SLE, and there was a negative correlation of Metrnβ with ILC2 as well. Further ROC analysis showed that the area under the curve (AUC) for Metrnβ was 0.8250 (95% CI: 0.7379–0.9121), with a cutoff value of 1131 pg/mL to effectively distinguish SLE patients from healthy controls. Our study herein demonstrated for the first time that Metrnβ values were increased and were immunologically correlated with SLE activity, which could be utilized as an alternative biomarker for the early identification and predicting of the immuno-response of SLE.

## 1. Introduction

Systemic lupus erythematosus (SLE) is a chronic autoimmune disorder affecting multiple organs, including the skin, joints, blood vessels and kidneys, etc. [1]. Often referred to as the prototype of systemic autoimmune illness, SLE predominantly affects women and is thought to be triggered by both genetic and environmental factors, such as UV light, infection, smoking and drugs, etc. [2,3]. However, the complex interplay between genetic and environmental factors in the pathogenesis of SLE has not been fully elucidated, making it a life-long pathogenic inflammatory condition with a prolonged diagnosis.

Extensive studies have demonstrated that SLE is initiated by immune-complex deposition and lymphocytic infiltration [4]. Both T-cells and B-cells have been found to exhibit hyperactivation throughout the progression of SLE, as a result of the excessive exposure of the immune system to self-antigens. Following their over-activation, T and B cells may interact with other immune cells like dendritic cells and macrophages, as well as inflammatory mediators like chemokines and cytokines, leading to the subsequent over-production of circulating autoantibodies against cellular components, including nuclear antigens, persistent autoimmune inflammation, and target organ damage [5,6]. For these reasons, identifying the function and underlying mechanisms of host inflammatory molecules during pathogenic inflammation in SLE may provide novel perspectives for its early diagnosis and treatment.

Metrnβ is a novel secreted cytokine/myokine first identified in 2012. Metrnβ was reported to be predominantly produced by activated macrophages and barrier tissues, and has been implicated in numerous diseases, ranging from metabolic disorders like diabetes and obesity [7], to inflammatory diseases like fulminant hepatitis, skin wound healing, psoriasis, prurigo nodularis, actinic keratosis, as well as regeneration [8,9,10]. Currently, the biologic functions of Metrnβ mainly focused on its roles as a myokine. For example, it has been reported that Metrnβ may enhance adipose tissue browning via mediating the STAT6 pathway of infiltrated macrophages in the tissue [11]. Metrnβ may also regulate adipocyte differentiation by activating PPARγ-mediated signaling, which subsequently increases insulin sensitivity [12]. Apart from working as a myokine, Metrnβ has also been recognized as a novel cytokine in recent years, and increased Metrnβ levels, which are crucial in balancing host inflammatory responses, have been observed in patients with COPD exacerbations [13]. Additionally, during muscle regeneration, a deficiency in Metrnβ failed to produce an anti-inflammatory phenotype [8]. Metrnβ^−/−^ mice can also exhibit dysregulated cytokine production, altered IgG synthesis, and increased susceptibility to sepsis [14], making Metrnβ a novel cytokine with anti-inflammatory properties. However, in terms of the investigation of Metrnβ in SLE, reports are not yet available. Given the broad immunoregulatory role of Metrnβ in inflammatory diseases, we herein hypothesized that Metrnβ may participate in the pathogenesis of SLE.

In this study, we sought to investigate whether Metrnβ can be a novel biomarker in the identification of SLE patients compared to healthy individuals, as well as to describe the immune microenvironment of SLE patients at the early phase of onset. To this end, we enrolled individuals clinically diagnosed with SLE and divided them into various groups based on their disease activity. We comprehensively analyzed the expression pattern and immunological correlation and predictive values of Metrnβ in SLE for the first time.

## 2. Results

### 2.1. Characteristics of the Recruited Patients and Healthy Controls

A total of 52 patients with SLE and a corresponding 53 healthy donors were enrolled. The demographics and clinical parameters are summarized in Supplementary Table S1. There was no significant difference regarding gender and age distribution between the two groups. SLE patients had significantly higher levels of Anti-dsRNA Ab, ESR and CRP compared to those of the healthy control (Supplementary Table S1). SLE patients were also classified into subgroups in accordance with the distinct activity based on their SLE-DAI index: no activity (inactive) (*n* = 13), mild (*n* = 19), moderate (*n* = 9), and high activity (*n* = 11).

### 2.2. Metrnβ Expressions and Their Association with SLE Disease Activity

**Figure 1:**
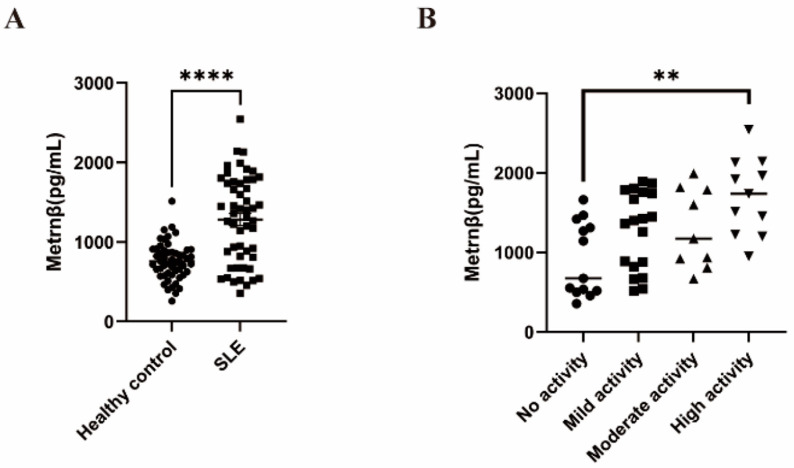
Metrnβ expression was elevated in patients with SLE. (**A**) Circulating Metrnβ concentrations in SLE patients (*n* = 52) and healthy controls (*n* = 53) were detected by ELISA. (**B**) SLE were stratified into no activity (*n* = 13), mild (*n* = 19), moderate (*n* = 9), and high activity (*n* = 11) groups based on their SLE-DAI scores, and serum Metrnβ levels were detected by ELISA. Data were presented as the mean ± SEM. The *p* values were calculated using the Mann–Whitney test, or the Kruskal–Wallis test followed by Dunn’s multiple comparisons post-test where appropriate. ** *p* < 0.01 and **** *p* < 0.0001.

As detected by ELISA, we found that there were significantly increased Metrnβ levels in clinically diagnosed SLE patients (1283 ± 543.4 pg/mL) compared to the healthy controls (759.9 ± 233.1 pg/mL) (Figure 1A). After dividing the SLE patients into inactive, mild, moderate and severe activity subgroups based on their SLE-DAI scores, we detected a gradual increase of Metrnβ levels with the progression of disease activity, and those with high activity (1707 ± 484.0 pg/mL) displayed significantly higher Metrnβ levels compared to the cases with no activity (912.6 ± 468.6 pg/mL) (Figure 1B). Together, the findings show that Metrnβ levels in SLE patients were significantly elevated, thereby indicating the potential regulatory roles of Metrnβ in SLE.

### 2.3. Association between Metrnβ and SLE Related Clinical Parameters

**Figure 2:**
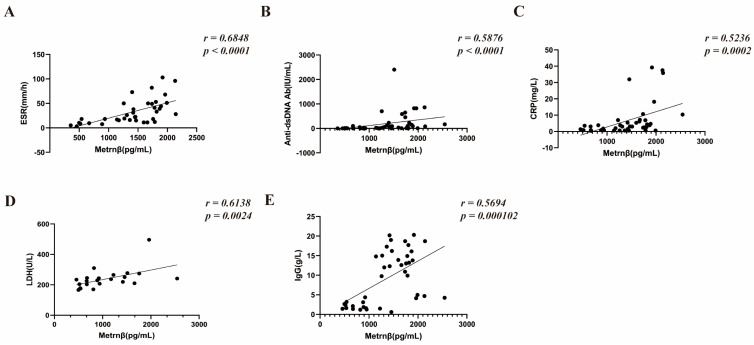
Correlation of Metrnβ with clinical parameters in patients with SLE. (**A**–**E**) Serum ESR (*n* = 37), Anti-dsDNA Ab (*n* = 47), CRP (*n* = 45), LDH (*n* = 22) and IgG (*n* = 41) in patients with SLE were routinely tested in the clinical laboratory, and Spearman’s correlation coefficient was used for their correlation analysis with serum Metrnβ levels. ESR: Erythrocyte Sedimentation Rate; CRP: C-reactive protein; LDH: Lactate dehydrogenase.

To further explore the possible regulatory role of Metrnβ in the pathogenesis of SLE, we tested whether serum Metrnβ levels correlated with SLE-related laboratory parameters. Of note, there was a positive correlation between circulating Metrnβ levels and ESR (*r* = 0.6848, *p* < 0.0001) (Figure 2A). Moreover, Metrnβ concentrations also showed a positive correlation with anti-dsDNA Ab (*r* = 0.5876, *p* < 0.0001), CRP (*r* = 0.5236, *p =* 0.0002), LDH (*r* = 0.6138, *p* = 0.0024), and total IgG concentrations (*r* = 0.5694, *p* = 0.000102) (Figure 2B–E), while there was no significant difference with regard to the concentration of circulating Metrnβ with C3 (*r* = −0.0424, *p* = 0.7823), C4 (*r* = −0.0478, *p* = 0.7552), IgA (*r* = −0.2890, *p* = 0.0542), IgM (*r* = −0.0723, *p* = 0.6533), urea (*r* = 0.1602, *p* = 0.2875) as well as creatine (*r* = 0.1408, *p* = 0.3506) (Figure S1). Together, these results provide further evidence that Metrnβ may participate in the pathogenesis of SLE.

### 2.4. Possible Regulations of Metrnβ on Immunological Tolerance during SLE Pathogenesis

**Figure 3:**
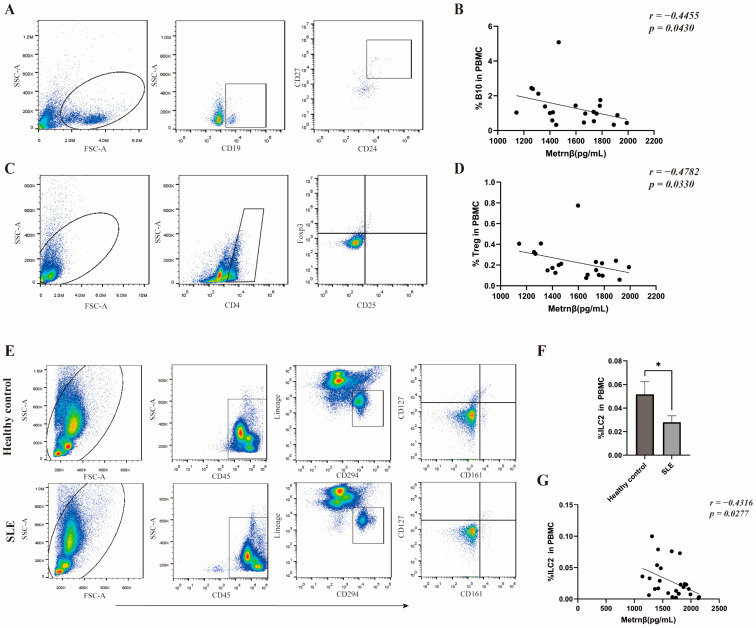
Correlation of circulating Metrnβ levels with peripheral immune cells of patients with SLE. (**A**–**D**) Peripheral B10 cells (defined as CD19^+^CD24^+^CD27^+^) and Tregs (defined as CD4^+^CD25^+^Foxp3^+^) were analyzed using flow cytometry. Representative graphs showing the gating strategies of B10 and Treg are displayed in (**A**,**C**), respectively. Circulating Metrnβ concentrations were detected by ELISA as described above. Association of Metrnβ with B10 (*n* = 21) and Tregs (*n* = 20) were performed thereafter and shown in (**B**,**D**), respectively. (**E**) Gating strategy and representative flow cytometric plot for the analysis of Lineage^−^CD45^+^CD294^+^CD127^+^CD161^+^ ILC2 cells in the peripheral blood of SLE patients. (**F**) Bar chart showing the percentage of ILC2 cells in the blood of SLE patients (*n* = 26) and healthy controls (*n* = 6) using flow cytometric analysis. (**G**) Correlation of serum Metrnβ levels with peripheral ILC2 cells (*n* = 26). Data were displayed as mean ± SEM. The difference between the two groups was obtained by conducting a Mann–Whitney test where appropriate. Spearman’s correlation coefficient was used for correlation analysis. * *p* < 0.05.

As an autoimmune disease, T and B cells in SLE contribute to the pathogenic inflammation and excessive auto-antibodies as reported [5]. In addition, SLE is characterized by the breakdown of immunological tolerance and homeostasis, in which regulatory T cells (Treg) as well as IL-10 producing B cells (B10) have been described as key regulators while deficit [15,16,17].We therefore compared the relationship of Metrnβ with these regulatory immune cells in SLE in order to further demonstrate the regulatory role of Metrnβ in SLE. As shown in Figure 3, there was an inverse correlation of Metrnβ with B10 (*r* = −0.4455, *p* = 0.0430) as well as Treg (*r* = −0.4782, *p* = 0.0330) (Figure 3B,D). Of note, Group 2 innate lymphoid (ILC2) cells, as T-helper type 2 (Th2)-related innate immune effector cells, have recently been shown to play protective roles in SLE by eliminating auto-reactive B cells [18]. In order to investigate whether the ILC2 may work for SLE, ILC2 cells were analyzed thereafter. We discovered that most SLE patients in the present study cohort showed decreased proportions of ILC2 in CD45^+^ peripheral blood cells (0.02805 ± 0.005371%) when compared to normal controls (0.05167 ± 0.01069%) (Figure 3F), and Metrnβ levels showed a negative correlation with ILC2 (*r* = −0.4316, *p* = 0.0277) (Figure 3G). Together, these results reveal that Metrnβ may possibly promote the initiation and progression of SLE via the disturbance of immunotolerance in SLE.

### 2.5. Association of Metrnβ with Distinct Inflammatory Patterns in SLE

**Figure 4:**
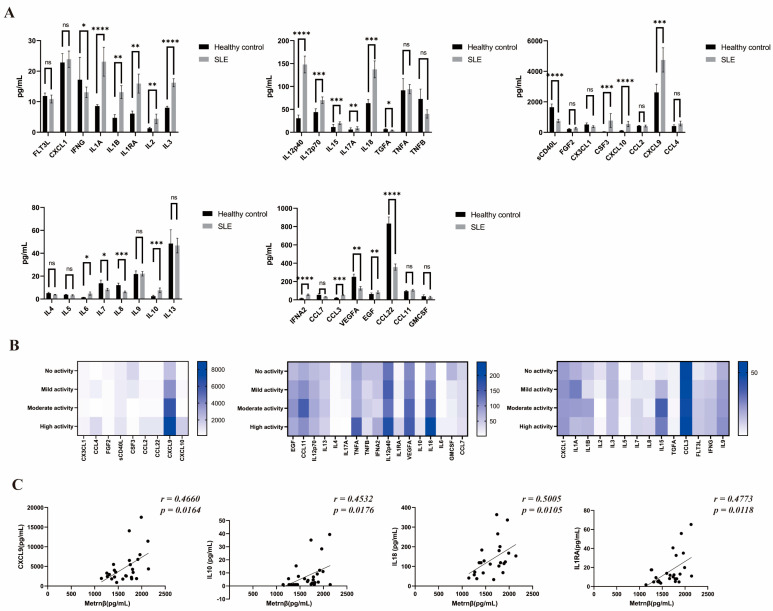
Disturbed immune response in SLE and promotional effect of Metrnβ on pathogenic inflammation during SLE. (**A**) Profiling of the cytokines/chemokines in SLE patients (*n* = 27) and paired healthy controls (*n* = 30) via Milliplex. (**B**) SLE patients were divided into no activity, mild, moderate and high activity subgroups based on SEI-DAI scores, and distinct cytokine/chemokine profiles in these groups were shown as heat maps. (**C**) Association analysis of circulating Metrnβ levels with CXCL9 (*n* = 26), IL10 (*n* = 27), IL18 (*n* = 23), and IL1RA (*n* = 27). Values were expressed as mean ± SEM. The difference between the two groups was determined by conducting a Mann–Whitney test. Spearman’s correlation coefficient was used for correlation analysis. * *p* < 0.05, ** *p* < 0.01, *** *p* < 0.001 and **** *p* < 0.0001. ns, non-significant difference.

It is widely recognized that pathogenic inflammation characterized by the elevation of a series of cytokines/chemokines contributes to the progression of SLE [19]. We then sought to evaluate the longitudinal change in cytokine/chemokine profiles of SLE patients after they were stratified by disease activity to further demonstrate the regulatory roles of Metrnβ in SLE. A significant increase of a wide range of inflammatory mediators were observed in SLE patients, including IL1A, IL1B, IL1RA, IL2, IL3, IL12p40, IL12p70, IL15, IL17A, IL8, CSF3, CXCL10, CXCL9, IL6, IL10, IFNA2, CCL3, EGF, while IFNG, TGFA, IL7, IL8, sCD40L, VEGFA and CCL22 were decreased in SLE patients compared to the healthy controls (Figure 4A). Among those inflammatory mediators, CXCL9, CXCL10, IL1RA, VEGFA, IL10, IL18, IL6, and IFNG in the high/moderate activity cases displayed higher expressions than the mild/no activity cases and the healthy control (Figure 4B). Of note, further correlation analysis revealed the possible association of Metrnβ with the disturbed immune response of SLE patients, as there was a positive correlation of Metrnβ with CXCL9 (*r* = 0.4660, *p* = 0.0164), IL10 (*r* = 0.4532, *p* = 0.0176), IL18 (*r* = 0.5005, *p* = 0.0105) and IL1RA (*r* = 0.4773, *p* = 0.0118) (Figure 4C). Overall, these findings provide further support for the positive correlation of Metrnβ with SLE related disease activity and pathogenic inflammation.

### 2.6. Potential Value of Metrnβ as a Biomarker in the Diagnosis of SLE

**Figure 5:**
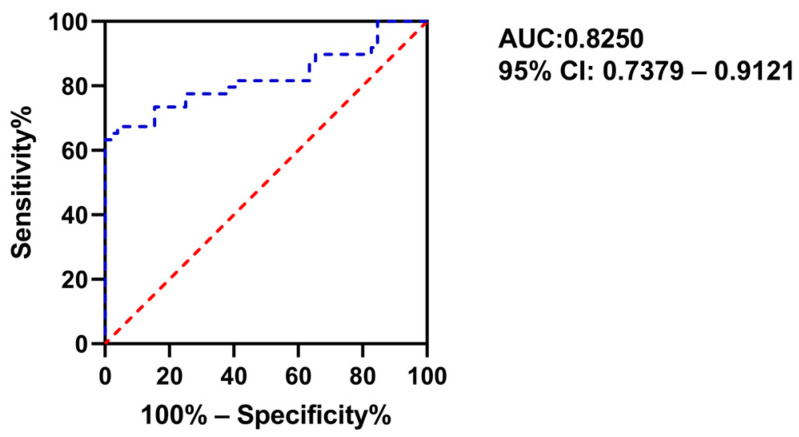
Diagnostic potential of Metrnβ in SLE. ROC was performed to assess the biomarker value of serum Metrnβ levels in distinguishing SLE patients (*n* = 52) from healthy controls (*n* = 53). The AUC was 0.8250 (95% CI: 0.7379–0.9121). The Youden index and best corresponding cutoff value were calculated based on the maximum value of sensitivity—(1-Specificity). AUC: area under the curve.

We speculate that serum Metrnβ may be a valuable biomarker in the early identification of SLE based on the substantially higher Metrnβ levels in the SLE patients compared to the healthy controls, and its positive correlation with disease activity as demonstrated above. Therefore, ROC analysis was performed for Metrnβ thereafter. As shown in Figure 5, we detected an AUC of 0.8250 (95%CI: 0.7379–0.9121) for Metrnβ to distinguish SLE patients from healthy individuals (Figure 5). Moreover, the cutoff value for Metrnβ to effectively distinguish SLE patients from healthy individuals was 1131 pg/mL, with a sensitivity at 67.35%, and a specificity at 96.15% (Table 1). As ESR, Anti-dsDNA Ab, CRP and LDH were increased in SLE patients both in our study and others, and these clinical parameters may also be indicated as biomarkers in SLE identification and severity [20,21,22,23], we also performed ROC analysis for ESR, Anti-dsDNA Ab, CRP, IgG and LDH so as to better compare the efficacy of Metrnβ with these markers. As shown in Table 1 and Figure S2, among the six parameters studied, the Metrnβ displayed the highest efficacy in differentiating SLE patients from normal controls. Together, the above results demonstrate that Metrnβ may be useful in guiding the early identification of SLE from healthy controls.

## 3. Discussion

Metrnβ is a novel secreted cytokine/myokine that has been implicated as immunoregulatory both in innate and acquired immunity. Current studies on Metrnβ have mainly focused on neurodevelopment, insulin resistance, beige fat thermogenesis, metabolism and inflammation related diseases [11,12,24]. To our knowledge, Metrnβ^−/−^ mice exhibit inflammatory lesions in the uterus, kidneys and liver, and have increased susceptibility to LPS-induced sepsis [14], thereby implicating Metrnβ as playing regulatory roles in the resolution of inflammation. Recently, Metrnβ has also been linked to the pathophysiology of autoimmune diseases such as rheumatoid arthritis [25] and psoriatic arthritis [26]. However, the potential regulatory roles of Metrnβ in SLE have not been investigated yet. In this study, we found elevated Metrnβ levels in SLE patients, and those with high activity displayed higher Metrnβ levels. We also showed that serum Metrnβ levels were positively correlated with clinical parameters like Anti-dsDNA Ab, ESR, LDH, CRP and total IgG, which are important risk factors and disease markers for SLE, suggesting that Metrnβ may be involved in the occurrence and development of SLE. To our knowledge, this is the first study assessing the change of Metrnβ levels and its associations with SLE activity.

SLE is a complex multisystem chronic inflammatory disease characterized by the breakdown of immune tolerance of B and T cells to self-antigens, the production of autoantibodies, as well as the dysregulation of cytokines and chemokines. During SLE, unrestricted B-cell hyperactivation and defective naive B-cell formation in germinal centers are caused by abnormal T-cell signaling and an imbalance in T-cell subsets. These abnormal adaptive immune system functions produced excessive amounts of autoantibodies, inflammatory responses, and end-organ damage of SLE as a result. B10 is a functionally defined regulatory B-cell subset that plays vital role in the control of inflammation and autoimmune diseases, although it is present at low numbers in peripheral blood [27]. Specifically, it has been reported that B10 may ameliorate the progression of SLE, similar to Treg [16,28]. In addition, it has been shown that ILC2, which is characterized by the release of type 2 cytokines including IL-5, IL-9, and IL-13, etc., regulates a number of immune responses and functions protectively in autoimmunity by inhibiting the synthesis of IL-27 and by generating IL-9 to interact with regulatory T cells [18,29]. In this study, we analyzed the frequencies of B10 and Treg in peripheral blood from patients with SLE, and discovered a downregulated ratio of B10, Treg and ILC2 that may contribute to the resolution of inflammation in SLE, which is in accordance with the reported studies [18,30,31,32]. Importantly, in our further studies, we found an inverse correlation between Metrnβ with B10 as well as Treg, indicating that Metrnβ may interfere with the disturbance of immunotolerance during SLE. In addition, the negative correlation of Metrnβ with ILC2 implicates that Metrnβ may also possibly promote SLE via downregulating on ILC2, while further in-depth ex vivo, in vitro and in vivo experiments are required to evaluate the detailed immunological regulations in terms of the impact of Metrnβ on immunotolerance and homeostasis in SLE.

In SLE, pathogenic inflammation arises from the overactivation of immune cells, but it may also promote T/B-cell activation and humoral immunity. In this study, we found that SLE patients displayed a disturbed immune microenvironment characterized by aberrant levels of cytokines. Notably, IL-10, an anti-inflammatory cytokine, increased whereas IL-5/13 remained unchanged in SLE, which was inconsistent with the changes in B10, Treg and ILC2 that may contribute to the resolution of inflammation. We believe that the production source, the distinct roles as the cause or result of the SLE development, as well as the regulatory roles they played in SLE as discussed above may possibly help explain this paradox in relation to those anti-inflammatory cells and cytokines. Given the inverse correlation of B10, Treg and ILC2 with SLE disease activity [18,30,31], it is possible that during the pathogenesis of SLE, the disease progression and development leads to the downregulation of the protective B10, Treg and ILC2. As for IL-10, although IL-10 serves as a general anti-inflammatory cytokine, it is predominantly produced by IL-10-secreting T cells during SLE to suppress the inflammation [33]. In combination with our finding that IL-10 was elevated in SLE patients, we speculate that IL-10 was produced as a result of pathogenic inflammation that developed in an attempt by the body to suppress the SLE related inflammation, namely, negative feedback. As for IL-5 and IL-13, the typical Type 2 cytokines which are also widely recognized as anti-inflammatory, studies have shown that IL-5/IL-13-secreting cell subsets were not significantly different between SLE patients and controls [33], which may help explain the unchanged IL-5/IL-13 levels between the SLE patients and healthy controls that we observed. Specifically, our study demonstrated a positive association of Metrnβ with inflammatory mediators like CXCL9, IL10, IL18 and IL1RA, lending further support to the claim that Metrnβ participates in the initiation and progression of pathogenic inflammation in SLE.

Together, given our current findings, we speculate that Metrnβ may participate in the pathogenesis of SLE through the disturbance of immune balance of SLE. On the one hand, Metrnβ may accelerate the pathogenic inflammation via downregulating on ILC2, B10 as well as Tregs, thereby promoting the imbalance between these immunoregulatory cells with pathogenic T and B cells, initiating a shift to the sustained increase of pathogenic T and B cells and production of the corresponding autoantibodies in SLE. On the other hand, Metrnβ may directly accelerate the production and secretion of inflammatory mediators like IL18 and IL-10, which may further promote the expansion and recruitment of pathogenic immune cells that drive the progression of SLE. We are waiting for future mechanism studies regarding the detailed regulatory roles and molecular mechanisms of Metrnβ in SLE.

Traditionally, clinical parameters like Anti-dsDNA Ab, CRP, LDH, ESR and total IgG levels are elevated and considered as probable biomarkers for SLE identification as well as disease activity and severity [20,21,22,23]. In this study, further evaluation of the diagnostic efficacy of these parameters by ROC demonstrated that Metrnβ had the best efficacy to distinguish SLE patients from healthy donors, with an AUC of 0.8250 and a cut-off value of 1131 pg/mL, suggesting that circulating Metrnβ may serve as a novel biomarker in SLE diagnosis and pathogenic inflammation.

Overall, our study investigated for the first time the potential regulatory roles played by Metrnβ in the pathogenesis of SLE and its value in distinguishing SLE patients from normal controls. Metrnβ is therefore considered a useful surrogate biomarker in the identification of SLE and pathogenic inflammation, due to its substantial increase in disease settings, its more pronounced levels in individuals with active conditions, and its positive association with disease activity. In such cases, anti-Metrnβ treatment could be considered a means of improving the outcome of the disease.

## 4. Materials and Methods

### 4.1. Study Design and Ethics

Patients with SLE (age ≥ 18 years) admitted to the affiliated hospital of Southeast University, Nanjing, China, from August 2022 to Jan 2023, and gender- and age- paired healthy donors were enrolled in our study. The exclusion criteria included more than 10 mg/day of prednisolone at the time of enrollment, previous B cell-depleting therapy, and the use of immunosuppressants. The diagnosis of SLE was conducted based on the American College of Rheumatology classification criteria for SLE (1997) [34]. Based on the systemic lupus erythematosus disease activity index (SLE-DAI) [35], SLE patients were stratified into the following subgroups: no activity (SLE-DAI = 0), mild activity (SLE-DAI = 1–5), moderate activity (SLE-DAI = 6–10), and high activity (SLE-DAI = 11–19). Subsequently, a total of 52 SLE patients and 53 healthy controls were enrolled in the study. The study was approved by the affiliated hospital of Southeast University Clinical Research Ethics Committee, and all eligible participants provided written informed consent according to the Declaration of Helsinki.

### 4.2. Quantification of Serum Metrnβ Levels

Serum from study populations was collected and diluted for 4 folds with sterile PBS. The concentration of serum Metrnβ was measured using an Enzyme-linked Immunosorbent Assay (ELISA) (R&D systems, Inc., Minneapolis, MN, USA) in accordance with the manufacturer’s instructions.

### 4.3. Longitudinal Cytokine/Chemokine Profile

A human cytokine Milliplex MAP assay kit was used for the quantification of human cytokines/chemokines via a Bio-Plex 200 system (Bio–Rad Laboratories, Hercules, CA, USA) that included: human soluble CD40 ligand (sCD40L), epidermal growth factor (EGF), fibroblast growth factor 2 (FGF2), FLT3L, C-X3-C motif chemokine ligand 1(CX3CL1), colony stimulating factor 3(CSF3), granulocyte macrophage colony-stimulating factor (GMCSF), Interferon alpha-2 (IF NA2), interferon gamma (IFNG), interleukin (IL)1A, IL1B, IL1RA, IL2, IL3, IL4, IL5, IL6, IL7, IL8/CXCL8, IL9, IL10, IL12p40, IL12p70, IL13, IL15, IL17A, IL18, C-C motif chemokine ligand (CCL)2, CCL3, CCL4, CCL7, CCL11, CCL22, C-X-C motif chemokine ligand (CXCL)1, CXCL9, CXCL10, transforming growth factor alpha (TGFA), tumor necrosis factor alpha (TNFA), tumor necrosis factor beta (TNFB), and vascular endothelial growth factor A (VEGFA). A total of 27 SLE patients and 30 corresponding sex- and age-matched healthy controls from the study population were available for cytokines and chemokines profiling. While some parameters were less than/exceeded the detection limit of the assay, the ultimate obtained cytokine/chemokines in each item were accordingly ≤27.

### 4.4. Flow Cytometry Analysis of Immune Cells

A total volume of 21mL of fresh venous blood obtained from 27 available SLE patients among the total study populations were collected, and a layer of peripheral blood mononuclear cells (PBMCs) were isolated via centrifuging in a 1.082 g/mL isotonic Percoll solution (GE Healthcare Life Sciences) for 25 min at 1800 r.p.m. Cell pellets were then stained immediately or cryopreserved for future staining using a monoclonal antibody specifically for CD45, CD4, CD19, CD24, CD25, CD27, CD294, CD127, CD161, Foxp3 and Lineage to define lymphoid populations including ILC2, B10 and Treg. During the process, cells from some samples lost viability, leading to insufficient cells being collected for flow cytometric analysis, which accounts for the obtained data ≤ 27. The cell populations were analyzed using an Attune NxT flow cytometer (Thermo Fisher, Waltham, MA, USA).

### 4.5. Measurement of Laboratory Parameters

The serum levels of C-reactive protein (CRP), lactate dehydrogenase (LDH), Erythrocyte Sedimentation Rate (ESR), Anti-dsDNA Ab, C3, C4, IgA, IgM, IgG, Urea and Creatine were assessed upon their administration during routine clinical laboratory services at the affiliated hospital of Southeast University.

### 4.6. Statistical Analysis

A nonparametric Mann–Whitney U test or a Kruskal–Wallis test followed by Dunn’s multiple comparisons post-test were applied for the comparison of the difference between groups. As for the correlation of Metrnβ with inflammatory mediators as well as laboratory parameters, Spearman’s rank correlation test was conducted. Receiver operating characteristic (ROC) curves were generated to evaluate the biomarker value of Metrnβ in SLE, in which the area under the curve (AUC), cutoff value, Youden index, corresponding sensitivity and specificity were calculated. All statistical analyses were performed using GraphPad Prism software (version 10.0; La Jolla, CA, USA), and 2-tailed *p* values < 0.05 were considered statistically significant.

## Figures and Tables

**Table 1:** AUC and Optimal Cutoff value of clinical parameters in the identification of SLE patients from healthy controls.

Parameters	AUC (95% CI)	Cutoff Value	Sensitivity %	Specificity %	Youden Index %
Anti-dsDNA Ab, IU/mL	0.8040 (0.7001–0.9078)	8.920	74.47	85.71	60.18
ESR, mm/h	0.6661 (0.5462–0.7860)	27.50	44.74	90.57	35.31
CRP, mg/L	0.8134 (0.7306–0.8962)	1.3	66.67	88.68	55.35
LDH, U/L	0.7215 (0.6032–0.8398)	161	100	35.85	35.85
IgG, g/L	0.5734 (0.4489–0.6979)	12.15	43.9	83.02	26.92
Metrnβ, pg/mL	0.8250 (0.7379–0.9121)	1131	67.35	96.15	63.50

## Data Availability

The data presented in this study are available on request from the corresponding author.

## Supplementary Materials

The following supporting information can be downloaded at: https://www.mdpi.com/1422-0067/24/17/13607/s1, Figure S1: Correlation of Metrnβ with clinical parameters in patients with SLE; Figure S2: Diagnostic value of clinical parameters in SLE; Table S1: Demographics and clinical parameters of patients with SLE and healthy controls.
