# Supplementary material for: Elevation of Metrnβ and Its Association with Disease Activity in Systemic Lupus Erythematosus
Source: Int J Mol Sci. 2023 Sep 2;24(17):13607. doi: 10.3390/ijms241713607 (PMC10563073; doi:10.3390/ijms241713607)
Supplement: Supplementary file 1 [file ijms-24-13607-s001.zip › ijms-2560776-supplementary.pdf]

Elevation of Metrnβ and its association with disease activity in Systemic lupus erythematosus

Chen Zhang [1,2,†], Shijie Cai [3,†], Ying Li [1], Xiaoyan Xu [4], Yonghui Liu [1], Huaiyu Qiao [1], Chun-Kwok Wong [5], Guoqiu Wu [1,3,] Hui Jin [2,6,*] and Xun Gao [1,3,*]

1 Center of Clinical Laboratory Medicine, Zhongda Hospital, Southeast University, Nanjing 210009, Jiangsu, China

2 Department of Epidemiology and Health Statistics, School of Public Health, Southeast University, Nanjing 210009, Jiangsu, China

3 Department of Laboratory Medicine, Medical School of Southeast University, Nanjing 210009, Jiangsu, China

4 Department of Rheumatology, Zhongda Hospital, Southeast University, Nanjing 210009, Jiangsu, China

5 Department of Chemical Pathology, Prince of Wales Hospital, The Chinese University of Hong Kong, Hong Kong, China

6 Key Laboratory of Environmental Medicine Engineering, Ministry of Education, School of Public Health, Southeast University, Nanjing 210009, Jiangsu, China

* Correspondence: xungao@seu.edu.cn (X.G.); jinhuihld@seu.edu.cn (H.J.)

† These authors contributed equally to the work.

Correspondence:

Dr. Xun Gao

Center of Clinical Laboratory Medicine

Zhongda Hospital

Southeast University

Nanjing, Jiangsu, PR, China

Tel: (86)18094241021; E-Mail: xungao@seu.edu.cn

Supplementary Table S1 Demographics and clinical parameters of patients with SLE and healthy controls

Characteristics	SLE patients (n=52)	Healthy controls (n=53)
Sex, female	52	53
Age, years	42.08±13.33	48.17±14.4
Anti-dsDNA Ab, IU/mL	195.5±410.5	4.499±4.296
ESR, mm/h	32.07±25.69	16.91±9.894
CRP, mg/L	6.272±10.06	0.9277±1.106
C3, g/L	0.8412±0.2704	0.6035±0.3683
C4, g/L	0.1979±0.08935	0.1644±0.1492
Urea, mmol/L	7.813±8.495	5.717±2.074
Creatine, µmol/L	93.98±122.0	60.15±14.35
LDH, U/L	236.3±67.90	186±51.59
IgA, g/L	7.365±6.191	3.368±2.606
IgG, g/L	9.231±6.775	7.003±4.997
IgM, g/L	0.7418±0.5840	0.7308±0.8011

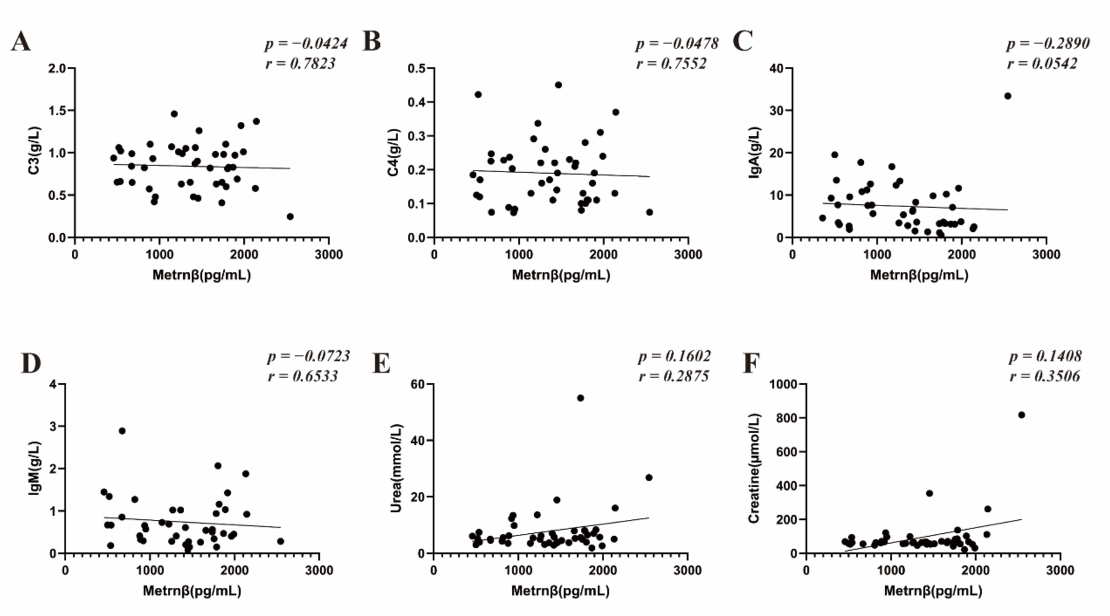

Figure S1. Correlation of Metrnβ with clinical parameters in patients with SLE. Serum C3 (n=45), C4 (n=45), IgA (n=45), IgM (n=41), Urea (n=46) and Creatine (n=46) in patients with SLE were routinely tested in the clinical laboratory, and Spearman's correlation coefficient was used for their correlation analysis with serum Metrnβ levels.

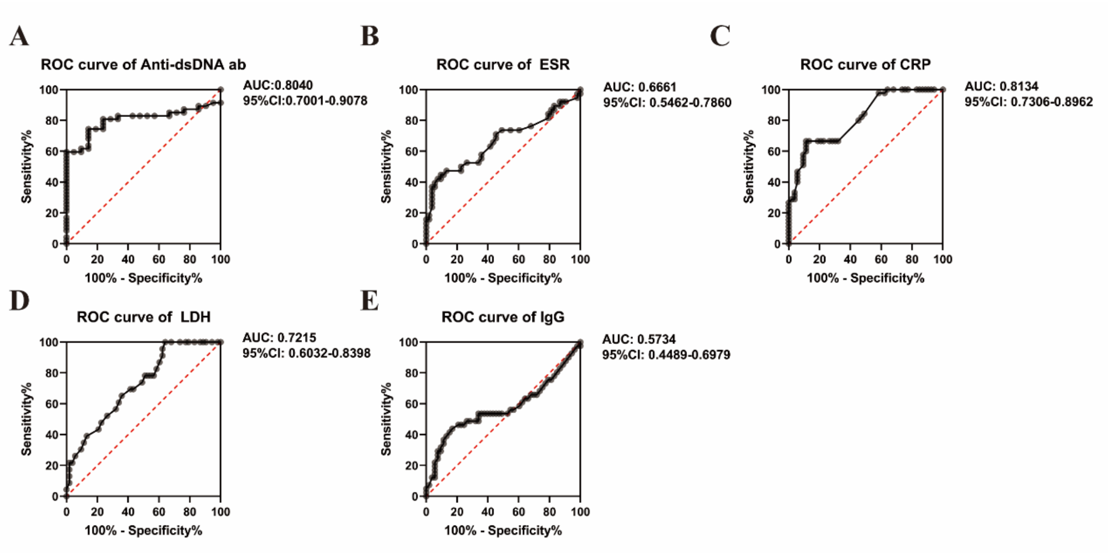

Figure S2. Diagnostic value of clinical parameters in SLE. ROC curve was displayed for analyzing the potential of Anti-dsDNA Ab (A), ESR (B), CRP (C), LDH (D) as well as IgG (E) in distinguishing SLE patients from healthy individuals. AUC: Area under the curve; ESR: Erythrocyte Sedimentation Rate; CRP: C-reactive protein; LDH: Lactate dehydrogenase.
